# Hesperetin inhibits foam cell formation and promotes cholesterol efflux in THP-1-derived macrophages by activating LXRα signal in an AMPK-dependent manner

**DOI:** 10.1007/s13105-020-00783-9

**Published:** 2021-07-02

**Authors:** Xuanjing Chen, Dezhi Zou, Xiaoling Chen, Huanlin Wu, Danping Xu

**Affiliations:** 1 Second Clinical Medical College, Guangzhou University of Chinese Medicine, Guangzhou, 510006 China; 2 Xiamen Hospital, Beijing University of Chinese Medicine, Xiamen, 361000 China; 3 Emergency Department, The First Affiliated Hospital, Sun Yat-Sen University, Guangzhou, 510080 China; 4 Cardiovascular Department, Guangdong Provincial Hospital of Chinese Medicine, 2nd Affiliated Hospital of Guangzhou University of Chinese Medicine, Guangzhou, 510120 China; 5 Guangdong Provincial Academy of Chinese Medical Sciences, Guangzhou, 510006 China; 6 Dongzhimen Hospital, Beijing University of Chinese Medicine, Beijing, 100700 China; 7 The Eighth Affiliated Hospital, Sun Yat-Sen University, Guangzhou, 510006 China

**Keywords:** Reverse cholesterol transport, Macrophage, Hesperetin, Lxrα, AMPK

## Abstract

Cholesterol efflux from macrophages is the first step of reverse cholesterol transport (RCT), whose increase inhibits cholesterol accumulation and foam cell formation to suppress atherogenesis. Hesperetin has been reported to exert several protective effects on cardiovascular diseases, while little is known about the role of hesperetin and its underlying mechanism in macrophage foam cell formation. In this study, we sought to investigate the potential effects of hesperetin on foam cell formation and cholesterol efflux by using human macrophages, focusing on liver X receptor alpha (LXRα) and AMPK. We found that hesperetin treatment reduced foam cell formation, intracellular cholesterol levels and the cholesterol esterification rate, and increased cholesterol efflux in THP-1 macrophages. Hesperetin increased the levels of LXRα protein and its targets, including ABCA1, ABCG1, SR-BI, and phosphorylated-AMPK. Meanwhile, the hesperetin-induced increase in LXRα expression was further increased by the AMPK agonist and inhibited by an AMPK inhibitor. Meanwhile, hesperetin increased the levels of LXRα mRNA and its target genes, all of which were decreased in cells transfected with the AMPKα1/α2 small interfering RNA (siRNA). Furthermore, the hesperetin-induced inhibition of foam cell formation and promotion of cholesterol efflux were decreased by transfection of AMPKα1/α2 siRNA. In conclusions, We are the first to report that hesperetin activate AMPK in THP-1-derived macrophages. This activation upregulats LXRα and its targets, including ABCA1, ABCG1 and SR-BI, which significantly inhibits foam cell formation and promotes cholesterol efflux. Our results highlight the therapeutic potential of hesperetin to possibly reduce foam cell formation. This new mechanism might contribute the anti-atherogenic effects of hesperetin.

## Introduction

Reverse cholesterol transport (RCT) is an effective approach to alleviate hypercholesterolemia and atherosclerosis [4]. It refers to the process by which excess cholesterol from lipid-laden peripheral cells is transferred to plasma high-density lipoprotein, then transported to the liver for bile acid synthesis, and finally excreted in the feces.

Liver X receptors (LXRs) are ligand-activated nuclear transcription factors comprising two isoforms, LXRα and LXRβ. LXRα is predominantly expressed in tissues and cells related to lipid metabolism, such as the liver, intestine, adipose tissue and macrophages [20]. LXRα plays a pivotal role in the transcriptional regulation of cholesterol homeostasis by regulating the transcription of genes that encode proteins involved in cholesterol biosynthesis, catabolism, secretion and reverse transport. Cellular cholesterol efflux, the initial step of RCT, is primarily mediated by three identified target proteins of LXRα, namely, ATP-binding cassette subfamily A member 1 (ABCA1), ATP-binding cassette subfamily G member 1 (ABCG1) and scavenger receptor class B type I (SR-BI) [29]. Activation of the LXRα signaling pathway by endogenous lipid ligands or synthetic agonists such as T0901317 promotes cellular cholesterol efflux from lipid-laden macrophages to attenuate atherosclerosis progression [15].

The transcriptional activity of LXRs, similar to other nuclear receptors, is regulated by posttranslational modifications, including acetylation, SUMOylation, and phosphorylation [16]. Previous studies have linked LXR phosphorylation to some kinases, such as mitogen-activated protein kinases, protein kinase C, casein kinase, and adenosine monophosphate-activated protein kinase (AMPK) [5, 26, 28]. AMPK is a ubiquitously expressed serine/threonine protein kinase complex that is responsible for maintaining balance between anabolic and catabolic pathways for cellular energy homeostasis. Once activated, AMPK triggers the catalytic process that generates ATP, which controls the biosynthesis and consumption of ATP [12]. Based on considerable evidence, AMPK activation inhibits the formation of foam cells and the deposition of atherosclerotic plaques [7, 16]. However, the association between AMPK and LXRα in a cellular model of atheroscleros is remains to be illustrated.

Flavonoid compounds possess multiple biological and pharmacological activities [3, 27]. Hesperetin, a citrus bioflavonoid, possesses prominent anti-inflammatory, anti-oxidant, anti-apoptosis, insulin-sensitizing and lipid-lowering activities, and exerts protective effects on the cardiovascular system, nervous system and the homeostasis of lipid and glucose metabolism [6, 8, 13]. Hesperetin reduces lipid accumulation in 3T3-L1 cells and rats liver [1, 14]. However, the effects of hesperetin on lipid accumulation in macrophages and the underlying mechanism remain largely obscure. Recently, hesperetin has been suggested as an enhancer of cholesterol efflux and a potent bioactivator of LXRα and AMPK [9, 24]. Based on these observations, we hypothesized that the enhancement of RCT by hesperetin inhibits foam cell formation and the LXRα and AMPK signaling pathway may be involved in this process. Therefore, we examined the effects of hesperetin on foam cell formation, cholesterol levels and cholesterol efflux in THP-1 macrophages. Furthermore, we investigated the underlying mechanisms regulating the levels of the LXRα mRNA and protein, as well as its downstream targets, and the role of AMPK in this process.

## Materials and methods

### Materials

RPMI-1640 medium, fetal bovine serum (FBS), and the BCA assay kit were purchased from Thermo Fisher Scientific (Waltham, MA, USA). Phorbol 12-myristate 13-acetate (PMA), T0901317 (293,754–55-9), Compound C (866,405–64-3), AICA-riboside (3031–94-5), 3-(4,5-dimethyl-2-thiazolyl)-2,5-diphenyl-2H-tetrazolium bromide (MTT) (298–93-1), Oil Red O (1320–06-5), and phenylmethanesulfonyl fluoride (PMSF) were purchased from Sigma-Aldrich (St. Louis, MO, USA). The Cholesterol Efflux Assay Kit (ab196985), anti-LXR alpha antibody (ab176323), anti-ABCA1 antibody (ab18180), anti-ABCG1 antibody (ab52617), and anti-scavenging receptor SR-BI antibody (ab217318) were purchased from Abcam (Cambridge, MA, USA). RIPA lysis buffer, the anti-phospho-AMPKα antibody (50,081), anti-β-actin antibody (4970), HRP-conjugated anti-rabbit IgG antibody (7074), and HRP-conjugated anti-mouse IgG antibody (7076) were purchased from Cell Signaling Technology (Danvers, MA, USA). Polyvinylidine difluoride membranes and the ECL kit (WBKLS0500) were purchased from Merck Millipore (Billerica, MA, USA). The control siRNA (sc-37007), AMPK alpha 1 siRNA (sc-29673), and AMPK alpha 2 siRNA (sc-38923) were purchased from Santa Cruz Biotechnology (Dallas, TX, USA). The Total RNA Extraction Reagent (R401-01), HiScript® II Q RT Super Mix (Q711-02/03), and ChamQ Universal SYBR qPCR Master Mix (Q711-02) were purchased from Vazyme Biotech Ltd. (Nanjing, China). The tissue total cholesterol assay kit (E1015) and tissue free cholesterol assay kit (E1016) were purchased from Applygen Technologies Inc. (Beijing, China). Oxidized low-density lipoprotein (ox-LDL), hesperetin (520–33-2), phosphatase inhibitor cocktail, and anti-AMPK alpha antibody (66,536–1-lg) were purchased from Yiyuan Biotechnologies (Guangdong, China), Chengdu Chroma-Biotechnology Ltd. (Sichuan, China), Roche (Basel, Switzerland), and Proteintech Group Inc. (Rosemont, USA), respectively.

### Cell culture and treatments

The THP-1 human monocytic leukemia cell line (SCSP-567) was obtained from Cell Bank of the Chinese Academy of Sciences (Shanghai, China). THP-1 cells were grown in RPMI-1640 medium supplemented with 10% FBS, 100 U/mL penicillin, 100 μg/mL streptomycin, 2.5 μg/mL amphotericin B and 2 mM L-glutamine in a humidified atmosphere containing 5% CO_2_ at 37 °C. Cells (1 × 10^6^ cells/mL) were cultured with 160 nM phorbol12-myristate 13-acetate for 24 h to facilitate their differentiation into macrophages. THP-1-derived macrophages were then incubated with 80 μg/mL ox-LDL combined with different compounds for another 24 h.

Reagents were prepared in a minimal volume of dimethyl sulfoxide (DMSO) and stored at -20 °C. For treatments, hesperetin (10–1000 μmol/L), T0901317 (10 μmol/L), AICAR (100 μmol/L) and Compound C (10 μmol/L) were added to the culture medium. The concentrations of reagents were determined considering the previous reports [30, 32].

### Cell viability

THP-1 cells were seeded in 96-well plates at a density of 1 × 10^4^ cells per well and cultured overnight. The cells were then treated with gradient dilutions of hesperetin (0–1000 μmol/L) for 24 h. Next, 5 mg/mL MTT in RPMI-1640 medium was added to each well of the plate and incubated with the cells at 37 °C for 4 h. Subsequently, the medium was discarded and cells were incubated with DMSO for 10 min at room temperature. The absorbance was measured at 570 nm with a microplate reader (BioTek Instruments Inc., Winooski, USA).

### Oil red O staining and analysis of lipid accumulation

THP-1 cells were seeded in 6-well plates at a density of 2 × 10^5^ cells per well and treated as described above. Subsequently, THP-1-derived macrophage foam cells were rinsed 3 times with cold phosphate-buffered saline (PBS) and then fixed with 4% paraformaldehyde for 30 min. After 3 rinses with distilled H_2_O, the cells were stained with 0.5 mL of a freshly filtered Oil Red O staining solution (0.5% Oil red O in 60% isopropanol) per well for 30 min at room temperature. Foam cells were observed and photographed using a light microscope (OLYMPUS BX53, Olympus, Tokyo, Japan).

After Oil Red O staining, intracellularly stained lipid droplets were extracted with isopropanol. The absorbance was measured at 492 nm with a microplate reader (BioTek Instruments Inc., Winooski, USA). The quantitative results were corrected after parallel experiments to determine cellular protein contents.

### Analysis of cellular cholesterol accumulation and cholesterol efflux

The cellular TC and FC levels were determined using the tissue total cholesterol assay kit and tissue free cholesterol assay kit. All experiments were performed according to the manufacturer’s instructions. The contents of cholesteryl ester (CE) were determined by subtracting the level of FC from the total cholesterol concentration. Cellular protein contents were calculated using a BCA assay kit.

The macrophage cholesterol efflux capacity was determined using a Cholesterol Efflux Assay Kit. The procedure was performed according to the manufacturer’s instructions. Briefly, THP-1 macrophages were pretreated with fluorescently labeled cholesterol for 6 h and then treated with or without hesperetin for 24 h. The cell supernatant was collected and cells were solubilized with cell lysis buffer. A microplate reader was used to analyze the absorbance of the supernatant and lysates at 490 nm. The percentage of cholesterol efflux was calculated as the ratio between the fluorescence intensity of the media and fluorescence intensity of the cell lysate plus media × 100%.

### Western blot analysis

Cells in 6-well plates were lysed with RIPA lysis buffer containing 1 mM PMSF and 1% of a phosphatase inhibitor cocktail (PhosSTOP, Roche group, Swiss). The supernatant was collected by centrifugation at 14,000 rpm for 15 min at 4 °C. The concentration of the cell lysate was determined using a Pierce BCA Protein Assay Kit. Equal amounts of proteins (60 μg each) were separated using 10% sodium-dodecyl sulfate polyacrylamide gel electrophoresis (SDS-PAGE) and transferred to polyvinylidine difluoride membranes at 300 mA for 1.5 h at 4 °C. Membranes were incubated with primary antibodies overnight at 4 °C after 1 h of blocking in 5% skim milk (Becton, Dickinson and Company, USA) at room temperature. Membranes were then washed with TBST for a total of 15 min and incubated with HRP-conjugated secondary antibodies for 1 h at room temperature. Protein bands were detected using an ECL kit, and band intensity was analyzed using Image Lab 5. 2. 1 software (Bio-Rad, Hercules, USA).

Primary antibodies included the anti-AMPK alpha antibody, anti-LXR alpha antibody, anti-ABCA1 antibody, anti-ABCG1 antibody, anti-scavenging receptor SR-BI antibody, and anti-β-actin antibody. Secondary antibodies included the HRP-conjugated anti-rabbit IgG antibody and HRP-conjugated anti-mouse IgG antibody.

### Transfection of small interfering RNAs

THP-1 macrophages were transfected with a nonsilencing control siRNA or siRNA targeting AMPKα1/α2 according to the manufacturer’s instructions. Briefly, THP-1-derived macrophages were seeded into 6-well plates at a density of 2 × 10^5^ cells/well. The siRNA duplex solution was added to the dilute transfection regent and incubated for 30 min at room temperature. After washing the cells once with transfection medium, cells were incubated with the transfection reagent mixture in transfection medium (siRNA final concentration at 60 nM) at 37 °C for 6 h. Then, 1 mL of RPMI-1640 medium containing 2 times the normal concentrations of serum and antibiotics was added to each wells and incubated with the cells for another 24 h. Subsequently, cells were treated with different reagents for 24 h. Target gene knockdown was validated using real time RT-PCR, and GADPH expression served as an internal control.

### Real-time RT-PCR analysis

THP-1 macrophages were seeded in 6-well plates. After transfection and treatments as described above, total mRNA was extracted using the Total RNA Extraction Reagent. First-strand cDNAs were synthesized from 1 μg of total RNA using a HiScript® II Q RT Super Mix. Then, 2 μL of cDNAs were amplified using the ChamQ Universal SYBR qPCR Master Mix in a total volume of 25 μL. Real-time quantitative polymerase chain reaction was conducted with a Bio-Rad CFX96 cycler and specific thermocycling conditions according to the manufacturer’s instructions. GADPH served as the internal control and quantitative measurements were analyzed using the ΔΔCt method. The primer sequences are presented in Table 1.

**Table 1:** Sequences of primer for real-time RT-PCR

Gene	Primer
GAPDH	F 5′-CAACGTGTCAGTGGTGGACCTG-3’
	R 5′-GTGTCGCTGTTGAAGTCAGAGGAG-3’
ABCA1	F 5′-CCCTGTGGAATGTACCTATGTG-3’
	R 5′-GAGGTGTCCCAAAGATGCAA-3’
LXRα	F 5′- GGATTTGGACAGTGCCTTGGT-3’
	R 5′-GTCAGGAGGAATGTCAGGCAC-3’
ABCG1	F 5′-CAGTCGCTCCTTAGCACCA-3’
	R 5′-TCCATGCTCGGACTCTCTG-3’
SRBI	F 5′-TTGCCAACGGGTCCATCTAC-3’
	R 5′-GAAACAAGGGGGCACTGAAC-3’
AMPKα1	F 5′-TGTAAGAATGGAAGGCTGGATGA-3’
	R 5′-GGACCACCATATGCCTGTGA-3’
AMPKα2	F 5′-GGTGATCAGCACTCCAACAGA-3’
	R 5′-TCTCTTCAACCCGTCCATGC-3’

### Statistical analysis

Statistical analysis was carried out with the help of Prism 7.0 software (Graphpad Software, CA, USA).

**Fig. 1:**
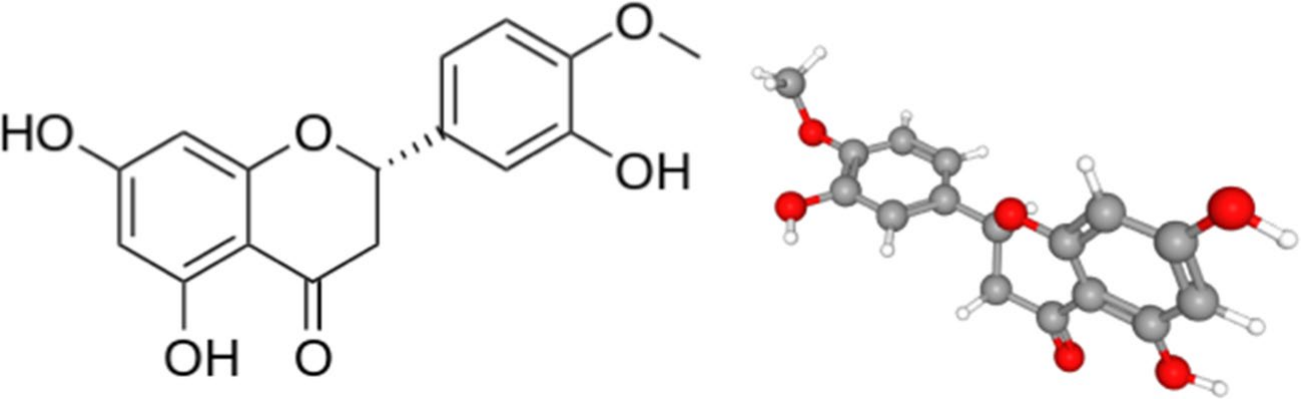
Structure of hesperetin

*P* values were calculated using ANOVA followed by Bonferroni and Tamhane’s T2 post-hoc analysis. Statistical significance was set at *P* < 0.05 (Fig. 1).

## Results

### Hesperetin inhibits foam cell formation and reduces lipid accumulation in THP-1 macrophages

**Fig. 2:**
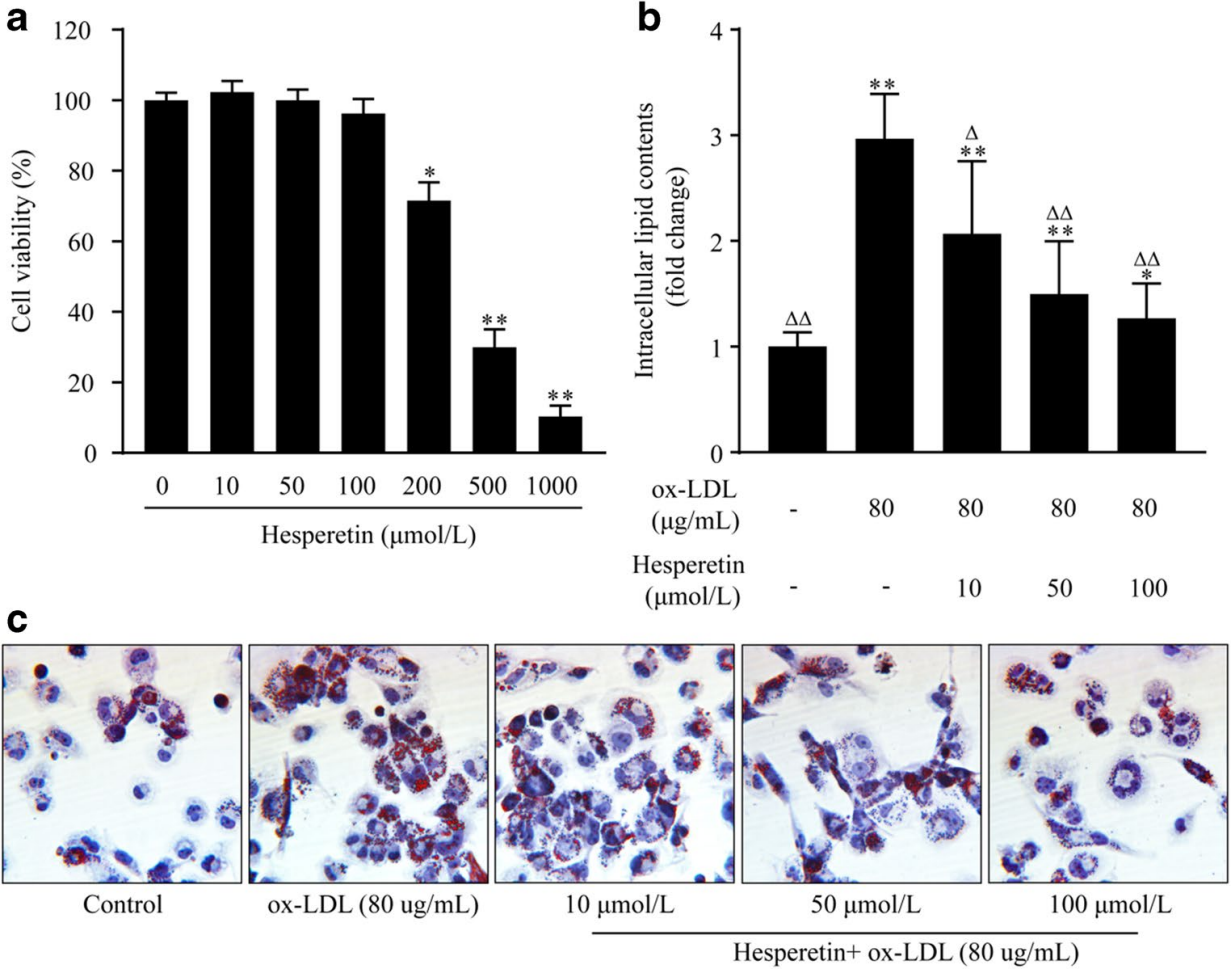
Effects of different hesperetin concentrations on the viability of THP-1 macrophages. (**a**) Cells were treated with gradient dilutions of hesperetin (0–1000 μmol/L) and subjected to cell viability experiments using the MTT method. The effect of hesperetin on lipid accumulation in THP-1 macrophage foam cells. THP-1 macrophages were treated with 80 μg/mL ox-LDL alone or in combination with hesperetin (10 μmol/L, 50 μmol/L and 100 μmol/L) for 24 h. Control cells were cultured for the same time without any treatments. (**b**) Representative microscopy images of Oil Red O-stained lipid droplets at a magnification of × 200. (**c**) Semiquantitative analysis of intracellular lipid contents based on the absorbance of intracellular dye extracted with isopropanol at 492 nm. The results are reported as the means ± SD from five independent experiments performed in triplicate. *p < 0.05 and ** *p* < 0.01 compared with control; Δ *p* < 0.05 and ΔΔ *p* < 0.01 compared with treatment with ox-LDL alone

First, we observed the effects of different concentrations (0–1000 μmol/L) of hesperetin on THP-1 macrophage activity. A high concentration of hesperetin decreased the viability of THP-1 macrophages, but a maximum hesperetin concentration of 100 μM did not affect cell viability (Fig. 2a). Therefore, we chose 10, 50 and 100 μmol/L concentrations of hesperetin to conduct the next experiments.

THP-1 macrophages were incubated with 80 μg/mL ox-LDL in the presence or absence of hesperetin for 24 h to investigate the effect of hesperetin on foam cell formation and lipid accumulation. Then, the Oil Red O-stained lipid droplets in cells were observed. Hesperetin significantly reduced the size and number of lipid droplets in THP-1 macrophage foam cells (Fig. 2c). A quantitative analysis of intracellular lipid contents was performed after the extraction of intracellular dye in isopropanol. Hesperetin reduced the lipid contents in THP-1 macrophage foam cells (Fig. 2b).

### Hesperetin reduces cholesterol accumulation and promotes cholesterol efflux in THP-1 macrophage foam cells.

**Fig. 3:**
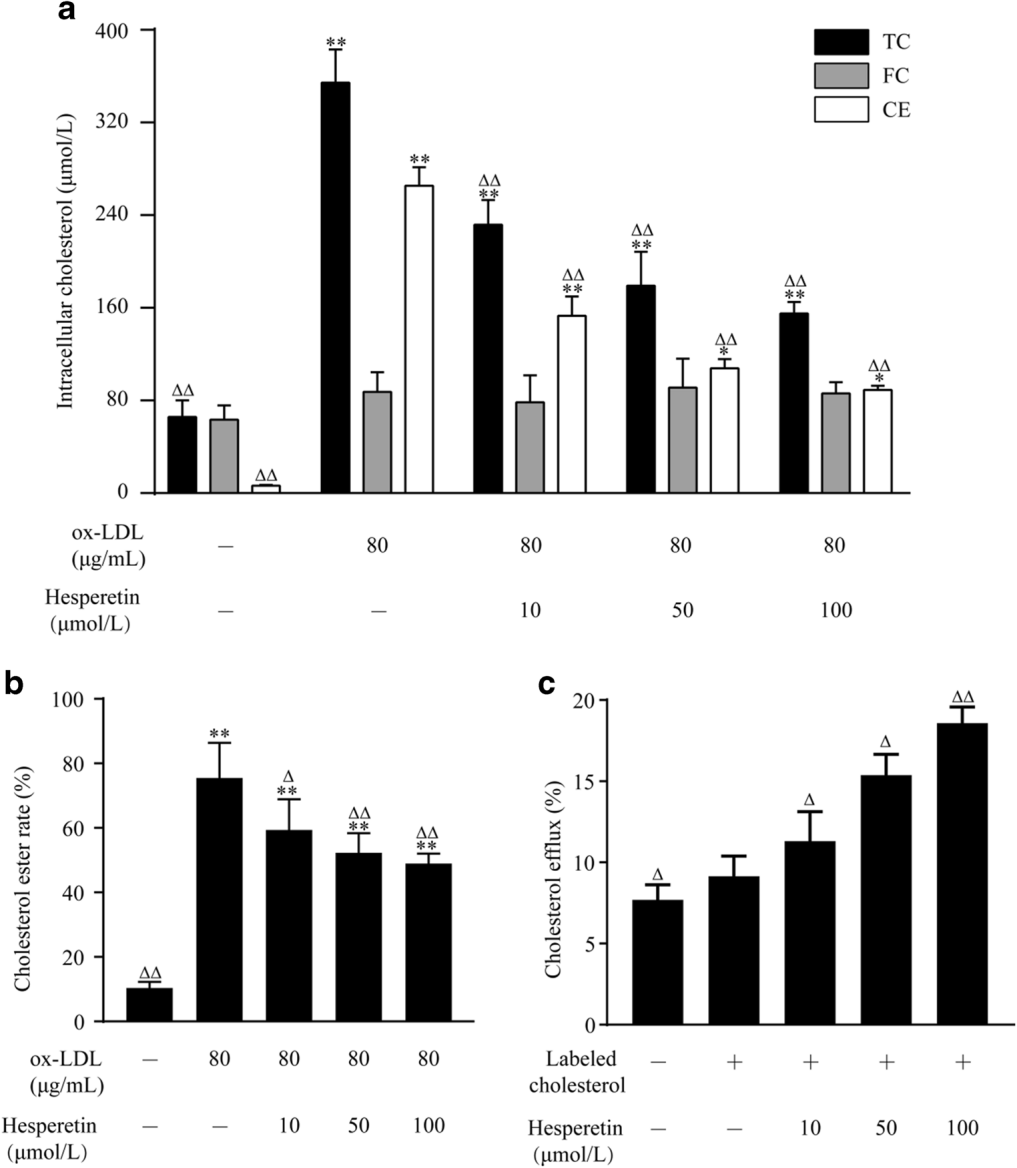
Effects of hesperetin on cholesterol accumulation and cholesterol efflux in THP-1 macrophage foam cells. THP-1 macrophages were treated with 80 μg/mL ox-LDL or labeled cholesterol alone or in combination with hesperetin (10 μmol/L, 50 μmol/L and 100 μmol/L) for 24 h. Control cells were cultured for the same time without any treatments. (**a**) The levels of total cholesterol (TC), free cholesterol (FC) and cholesterol ester (CE) in THP-1 macrophage foam cells were measured using an enzymatic method. (**b**) The cholesterol esterification rate (CER) is calculated as the ratio of CE to TC levels. (**c**) A commercial assay was used to determine cholesterol efflux as described above. The results are presented as the means ± SD from five independent experiments each performed in triplicate. *p < 0.05 and ** *p* < 0.01 compared with control; Δ *p* < 0.05 and ΔΔ *p* < 0.01 compared with treatment with ox-LDL or labeled cholesterol alone

Since hesperetin exerted a significant effect on reducing lipid accumulation, we postulated that hesperetin would also inhibit cholesterol accumulation. As shown in Fig. 3a and b, compared with control cells, total cholesterol (TC) levels, cholesterol ester (CE) levels, and the esterification rate (CE/TC) were significantly increased after treatment with ox-LDL. However, hesperetin reduced intracellular TC and CE levels, as well as the esterification rate.

Furthermore, we assessed cholesterol efflux to investigate how hesperetin reduces the intracellular cholesterol level. As shown in Fig. 3c, hesperetin increased cholesterol efflux to 11.31%, 15.38% and 18.58% at concentrations of 10 μmol/L, 50 μmol/L, and 100 μmol/L, respectively, all which were significantly different from cells treated with ox-LDL alone.

### Hesperetin upregulates the expression of the LXRα protein and mRNA and LXRα target proteins in THP-1 macrophages.

**Fig. 4:**
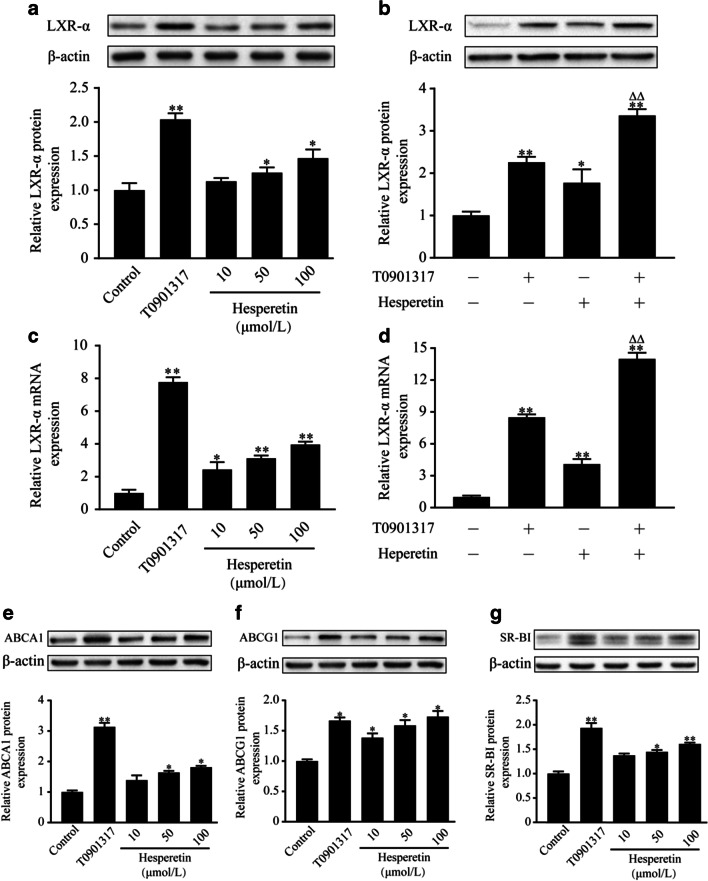
Effects of hesperetin on LXRα signal in THP-1 macrophages. THP-1 macrophages were treated with hesperetin (10 μmol/L, 50 μmol/L, 100 μmol/L) and/or T0901317 (10 μmol/L) for 24 h. Control cells were cultured for the same time without any treatments. Total proteins and RNAs were extracted and analyzed using western blot and real-time RT-PCR, respectively. (**a** and **c**) The effects of hesperetin on the levels of the LXRα protein and mRNA. (**b** and **d**) The best effective concentration of hesperetin of 100 μmol/L was used to further validate the hesperetin-induced activation of LXRα. (**e–g**) The effects of hesperetin on the levels of the ABCA1, ABCG1, and SR-BI proteins. The results are presented as the means ± SD from three independent experiments performed in triplicate. * *p* < 0.05 and ** *p* < 0.01 compared with control; Δ *p* < 0.05 and ΔΔ *p* < 0.01 compared with treatment with T0901317

LXRα signaling plays a critical role in cellular cholesterol homeostasis. Therefore, we assessed the effects of hesperetin on levels of the LXRα protein and mRNA. As shown in Fig. 4a-d, hesperetin increased the levels of the LXRα protein and mRNA, while this increase was lower than in cells treated with T0901317. We chose the best effective concentration of hesperetin at 100 μM to further validate its role in activating LXRα. Interesngly, when cells were treated with hesperetin and T0901317, a accumulative effects on the levels of the LXRα protein and mRNA was observed, which was a significantly different compared to cells treated with T0901317 alone.

We further examined the role of hesperetin in modulating the levels of LXRα downstream target proteins, including ABCA1, ABCG1, and SR-BI. Hesperetin increased the levels of the ABCA1, ABCG1 and SR-BI proteins. Among these proteins, ABCG1 levels were increased by hesperetin to a slightly greater extent than by T0901317. However, the increases in the levels of ABCA1 and SR-BI were lower than the increases induced by T0901317 (Fig. 4e-g).

### AMPK is implicated in hesperetin-induced LXRα activation.

**Fig. 5:**
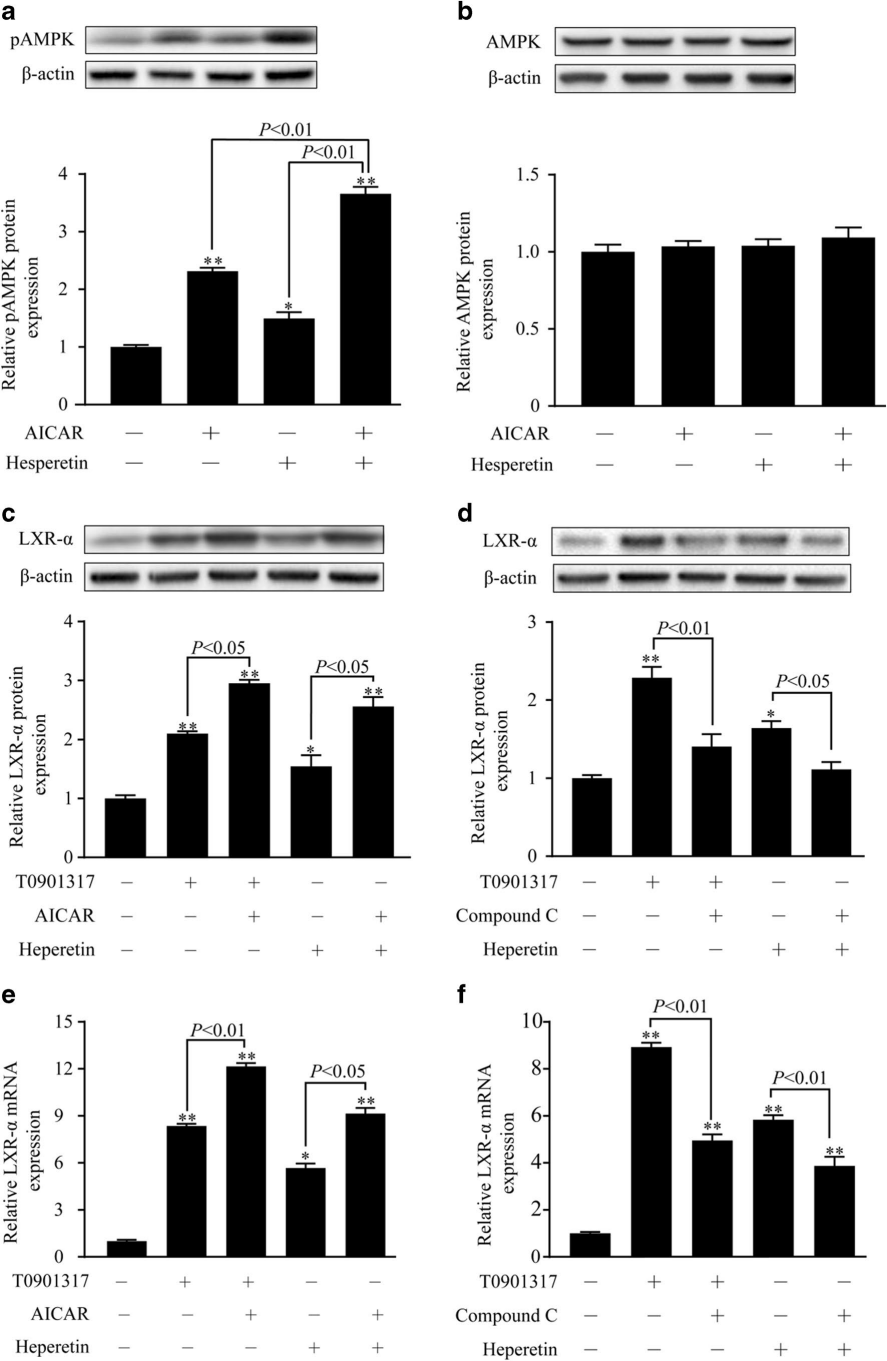
The role of AMPK in hesperetin-induced LXRα activation in THP-1 macrophages. THP-1 macrophages were treated with hesperetin (100 μmol/L) or T0901317 (10 μmol/L) in the presence or absence of AICARA or Compound C. Control cells were cultured for the same time without any treatments. Total proteins and RNAs were extracted and analyzed using western blot and real-time RT-PCR, respectively. (**a** and **b**) The levels of the phosphorylated AMPK and total AMPK proteins. (**c** and **e**) The effects of AMPK activation on the increase in the levels of the LXRα protein and mRNA induced by T0901317 or hesperetin. (**d** and **f**) The effects of AMPK inhibition on the increase in the levels of the LXRα protein and mRNA induced by T0901317 or hesperetin. The results are presented as the means ± SD from three independent experiments performed in triplicate. *p < 0.05 and ** *p* < 0.01 compared with control

AMPK has been described as a key regulator of lipid synthesis. Therefore, the effect of hesperetin on AMPK activation was examined. As shown in Fig. 5a, hesperetin significantly increased the level of the phosphorylated AMPK (pAMPK) protein by 1.5-fold compared with the control group of THP-1 macrophages. Meanwhile, a accumulative effect was observed when cells were treated with the combination of AICAR and hesperetin. In cells administered this treatment, the level of the pAMPK protein are significantly increased 1.5-fold compared with the cells treated with AICAR alone. However, a statistically significant difference in total levels of the AMPK protein was not observed between groups (Fig. 5b).

LXRα has been reported to be regulated by the phosphorylation of AMPK. We therefore investigated whether the activation of AMPK induced by hesperetin effectively induces LXRα activation. The agonist (AICAR) and inhibitor (Compound C) were incubated with cells. AICAR increased the levels of the LXRα protein in the cells stimulated with T0901317 or hesperetin (Fig. 5c). Meanwhile, Compound C decreased the levels of the LXRα protein in cells stimulated with T0901317 or hesperetin (Fig. 5d). Similar results were obtained for the mRNA level (Fig. 5e and f).

### Transfection of the AMPKα1/α2 siRNA decreased hesperetin-induced activation of LXRα signal, inhibition of foam cell formation, and promotion of choleterol efflux

**Fig. 6:**
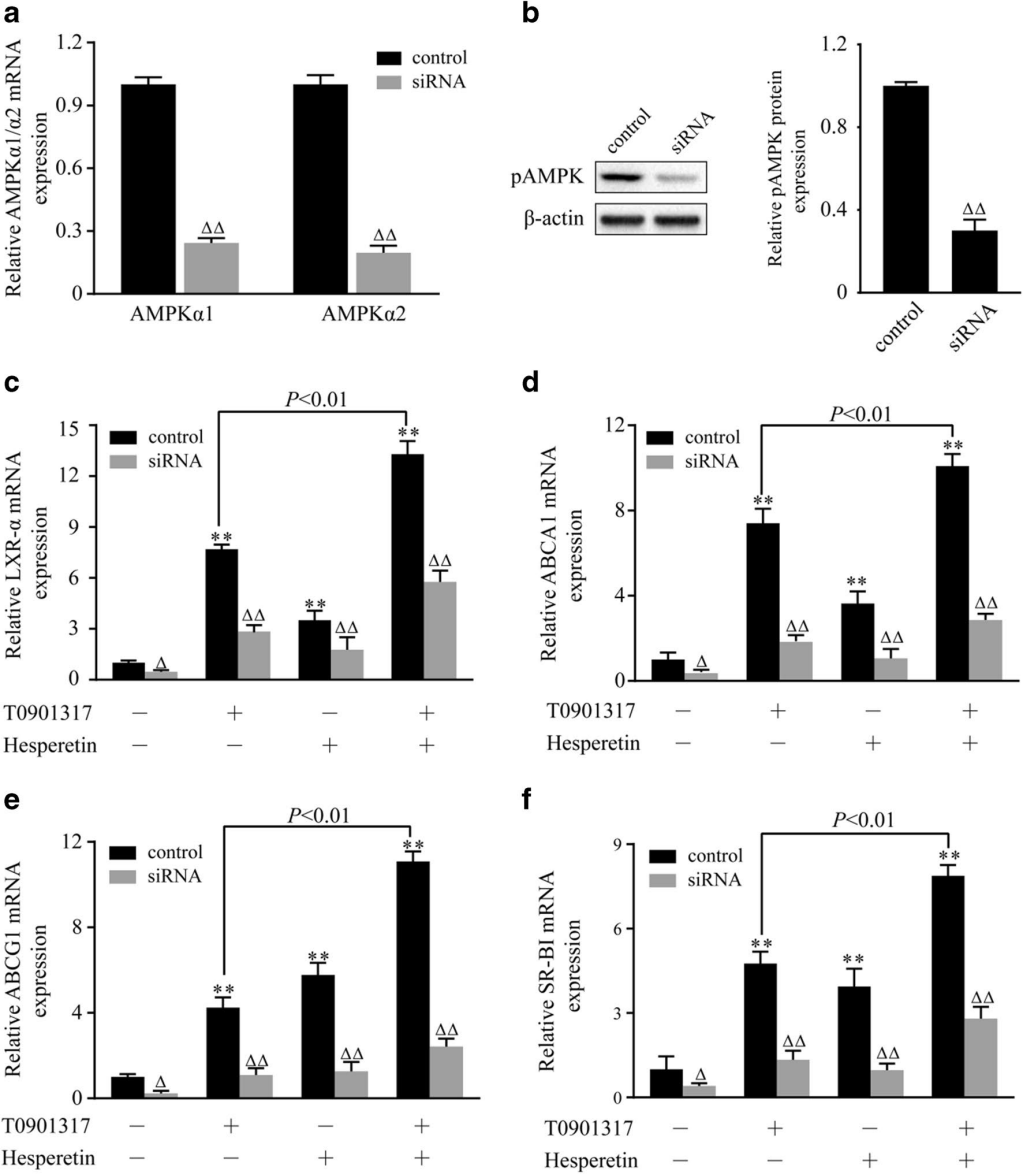
Effects of the AMPKα1/α2 siRNA on hesperetin-induced increases in the expression of LXRα and its target genes. THP-1 macrophages were transfected with the control siRNA or siRNA targeting AMPKα1/α2. Then, the cells were treated with hesperetin (100 μmol/L) and/or T0901317 (10 μmol/L). Control cells were cultured for the same time without any treatments. (**a**) The expressions of the AMPKα1, AMPKα2 mRNAs. (**b**) The expression of the pAMPK protein. (**c**-**f**) The expressions of the LXRα, ABCA1, ABCG1, and SR-BI mRNAs. The results are reported as the means ± SD from three independent experiments performed in triplicate. *p < 0.05 and ** *p* < 0.01 compared with control; Δ *p* < 0.05 and ΔΔ *p* < 0.01 AMPKα1/α2 siRNA vs. control siRNA

**Fig. 7:**
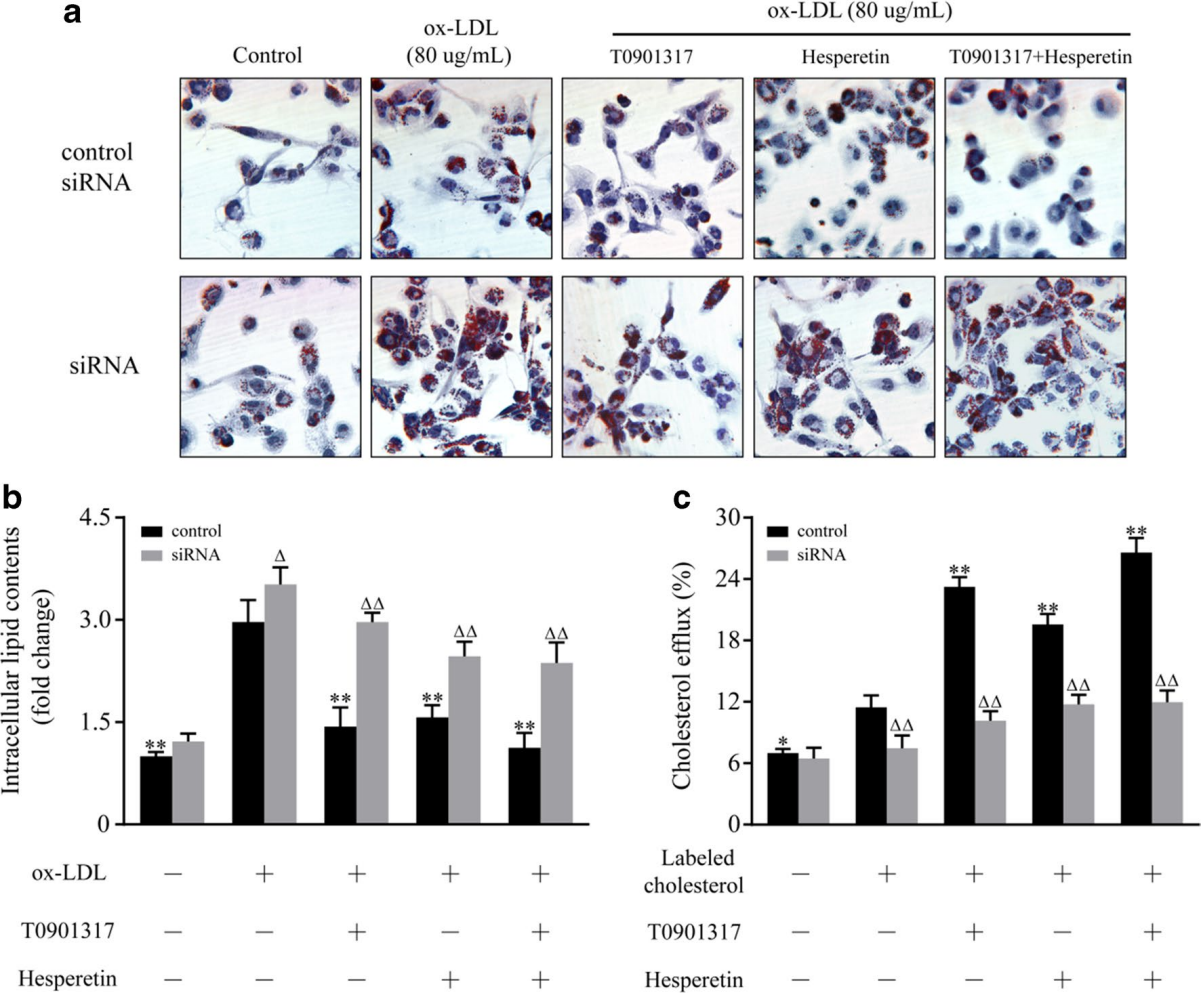
Effects of the AMPKα1/α2 siRNA on hesperetin-induced inhibition of foam cell formation and promotion of cholesterol efflux. THP-1 macrophages were transfected with the control siRNA or siRNA targeting AMPKα1/α2. Then, the cells were treated with 80 μg/mL ox-LDL or labeled cholesterol alone or in combination with hesperetin (100 μmol/L) and/or T0901317 (10 μmol/L) for 24 h. Control cells were cultured for the same time without any treatments. (**a**) Representative microscopy images of Oil Red O-stained lipid droplets at a magnification of × 200. (**b**) Semiquantitative analysis of intracellular lipid contents based on the absorbance of intracellular dye extracted with isopropanol at 492 nm. (**c**) The cholesterol efflux was determined by a commercial assay as described above. *p < 0.05 and ** *p* < 0.01 compared with treatment with ox-LDL or labeled cholesterol alone; Δ *p* < 0.05 and ΔΔ *p* < 0.01 AMPKα1/α2 siRNA vs. control siRNA

THP-1 macrophages were transfected with a specific AMPKα1/α2 siRNA to further validate the role of AMPK in the hesperetin-induced upregulation of LXRα and its target genes. A control siRNA that will not lead to a specific decrease in mRNA expression was used as a control (Fig. 6a). In control siRNA group, hesperetin significantly increased the levels of the LXRα mRNA and its downstream targets including ABCA1, ABCG1 and SR-BI. Meanwhile, the accumulative effects were observed when cells were treated with the combination of T0901317 and hesperetin (Fig. 6c-f). In line with this result, the accumulative effects were also observed in intracellular lipid contents and cholesterol efflux when cells were treated with T0901317 combined with hesperetin (Fig. 7a-c).

After transfection with the AMPKα1/α2 siRNA, the expression of the AMPKα1 and AMPKα2 mRNAs and the level of the pAMPK protein were significantly decreased (Fig. 6a and b). The transfection of this siRNA also reduced the expression of the LXRα mRNA and its target genes, including ABCA1, ABCG1 and SR-BI, compared with cells transfected with the control siRNA. Similar changes were observed in cells treated with T0901317 or hesperetin (Fig. 6c-f). In line with these results, transfection of the AMPKα1/α2 siRNA increased the lipid contents and inhibited the cholesterol efflux in THP-1 macrophage foam cells (Fig. 7a-c). Meanwhile, the effects of hesperetin and/or T0901317 on foam cell formation and cholesterol efflux were almost abolished by transefection of AMPKα1/α2 siRNA (Fig. 7a-c). These outcomes suggest that AMPK is implicated in hesperetin-induced activation of LXRα signal and LXRα signal-mediated inhibition of foam cell formation and cholesterol efflux.

## Discussion

In present study, we showed for the first time that hesperetin activated AMPK in THP-1 macrophages. This activation upregulated LXRα and its downstream targets, including ABCA1, ABCG1 and SR-BI, to significantly inhibit foam cell formation, reduce lipid and cholesterol accumulation, and promote cholesterol efflux in THP-1 macrophages.

The early stages of atherosclerosis are characterized by lipoprotein cholesterol accumulation and foam cell formation within blood vessel walls [4]. The incidence of cardiovascular events in patients enrolled in the Dallas Heart Study was inversely correlated with cholesterol efflux [22]. According to numerous studies, hesperetin possesses hypolipidemic, anti-inflammatory and endothelial cell-protective properties [10, 21]. As shown in a recent study by Noriko Sugasawa and colleagues, hesperetin evidently attenuates the development of atherosclerotic lesions in apolipoprotein E knockout mice [25]. However, the potential cytotoxicity of some flavonoids have been reported especially at high concentrations resulted from its pro-oxidant activity and mitochondrial toxicity [19]. In the present study, we first examined the effects of different concentrations (0–1000 μmol/L) of hesperetin on THP-1 macrophage activity to avoid its potential cytotoxicity at high concentration. Then the concentrations at 10, 50, 100 μmol/L of hesperetin without toxic effects to THP-1 macrophages were selected for next experiments. We founded that hesperetin increased cholesterol efflux from THP-1 macrophages, and reduced foam cell formation, cholesterol accumulation and cholesterol esterification in THP-1 macrophages, which might contribute to the observed anti-atherogenic and hypolipidemic effects of hesperetin.

LXRα has consistently been shown to be a promising anti-atherogenic target, due to its roles in inhibiting inflammation and promoting reverse cholesterol transport [15]. Activation of LXRα induces the expression of ABCA1, ABCG1 and SR-BI, all of which are membrane pumps exporting cholesterol to extracellular acceptors. Hesperetin has been reported to upregulate the enhancer activity of LXR and the expression of the ABCA1 mRNA in THP-1 macrophages [9]. The findings from our study are consistent with these results and added the findings that hesperetin significantly upregulated the expression of the LXRα mRNA and its target genes ABCG1 and SR-BI. These changes were validated at the protein level. Our findings provide two new targets, ABCG1 and SR-BI, for the effects of hesperetin on foam cells formation and cholesterol efflux. Meanwhile, unlike the preferential upregulation of ABCA1 to ABCG1 by an LXRα agonist, hesperetin upregulated the expression of these two transporters to a similar level. Thus, in addition to LXRα signaling, trans-activating factors specifically control the transcriptional regulation of ABCG1 in these cells. Additionally, the accumulative effect of combined T0901317 and hesperetin on LXRα signaling was observed. Similar effects have been reported for other flavonoids, such as the accumulative effect of combined T0901317 and curcumin on cholesterol efflux and the levels of ABCA1 mRNA and protein in THP-1 derived foam cells, and the upregulation of LXRα mRNA induced by resveratrol combined with T0901317 [17, 23].

Phosphorylation of AMPK has been shown to upregulate LXRα expression [2]. Some of flavonoids were considered the effective therapeutic approaches to modulate AMPK activity [11, 31]. According to a recent study by Hajar Shokri Afra1 et al., AMPK phosphorylation, an indicator of AMPK activation, was enhanced by hesepretin in HepG2 cells [24]. In the present study, we founded for the first time that hespretin increased the phosphoralated level of AMPK in THP-1 macrophages. Meanwhile, an enhancement in phosphorylation of AMPK protein after the conjoint treatment of hesperetin and AICAR was detected, which is similar to the effect of hesperetin combined with T0901317 on LXRα signal. Analogous effects have been reported with other flavonoids [11, 31]. These results revealed a clear effect of hesperetin on AMPK activation in THP-1 macrophages. Based on the multiple biological functions of AMPK signaling, we suggested that hesperetin might be a promising therapeutic strategy targeting the activated AMPK signaling pathway in various pathological processes.

We next examined the role of AMPK in hesperetin-induced LXRα activation. Hesperetin or T0901317 were administered in combination with or without the AMPK synthetic agonist (AICAR) or inhibitor (Compound C) to THP-1 macrophages. Then, the changes in LXRα expression were observed. Hesperetin-induced LXRα activation was enhanced by AICAR and inhibited by Compound C. Similar results were observed in cells treated with T0901317. Furthermore, transfection with the specific AMPKα1/α2 siRNA was performed to reduce phosphorylation of AMPK, which validated the role of AMPK in regulating the expression of LXRα and its target genes. Cells transfected with the AMPKα1/α2 siRNA displayed significantly reduced hesperetin and/or T0901317-induced upregulation of the LXRα mRNA and its targets genes, including ABCA1, ABCG1, and SR-BI. Meanwhile, the hesperetin and/or T0901317-induced inhibition of foam cell formation and promotion of cholesterol efflux were almost abolished by the siRNA transfection targeting AMPKα1/α2. We therefore conclude that AMPK is implicated in hesperetin-induced activation of LXRα signal and LXRα signal-mediated inhibition of foam cell formation and promotion of cholesterol efflux. Our results were consistent with previous observations that AMPK activated LXRα in human macrophages [17]. However, AMPK has also been reported to inhibit LXRα activation to prevent lipid accumulation in HepG2 cells [18]. This discrepancy may be due to the different characteristics of pathological cell models, suggesting that AMPK may selectively regulate LXRα activation in different pathological process.

In conclusion, hesperetin prevents foam cell formation, reduces total cholesterol levels and the cholesterol esterification rate, and increases cholesterol efflux in THP-1 macrophages through the activation of LXRα signaling pathway in an AMPK-dependent manner. This new mechanism contributes to the knowledge of the beneficial effects of hesperetin on regulating cholesterol homeostasis. We postulate that similar to other flavonoids, hesperetin will naturally find its place in the routine treatment of hyperlipemia and atherosclerosis.

## Data Availability

The datasets used and/or analyzed during the currentstudy are available from the corresponding author on reasonable request.

## Abbreviations

ABCA1: ATP-binding cassette subfamily A member 1
ABCG1: ATP-binding cassette subfamily G member 1
ABCG5: ATP-binding cassette subfamily G member 5
AMPK: Adenosine monophosphate-activated protein kinases
CE: Cholesterol ester
CER: Cholesterol esterification rate
FC: Free cholesterol
LXRα: Liver X receptor alpha
ox-LDL: Oxidized low-density lipoprotein
pAMPK: Phosphorylated AMPK
RCT: Reverse cholesterol transport
siRNA: Small interfering RNA
SR-BI: Scavenger receptor class B type I
TC: Total cholesterol
